# The paradoxical extinction: Exploring signatures of assortative mating as a possible mechanism that maintains canonical Red Wolf genetic ancestry in the American Gulf Coast canids

**DOI:** 10.1093/jhered/esag010

**Published:** 2026-01-30

**Authors:** Bridgett M vonHoldt, India Macaire, Kristin E Brzeski

**Affiliations:** Ecology and Evolutionary Biology, Princeton University, 08544 Princeton, NJ, United States; Department of Biology, University of Oxford, OX1 3SZ, Oxford, England, United Kingdom; College of Forest Resources and Environmental Science, Michigan Technological University, Houghton, MI, United States

**Keywords:** assortative mating, canids, genomic ancestry, simulation

## Abstract

Admixed genomes, particularly those with an evolutionary history of genetic exchange with an endangered or extinct species, are valued for innovative and unconventional conservation actions. Here, we show the substantial conservation value that the admixed canids of the Gulf Coast have as they retain high amounts of contemporary Red Wolf ancestry and unique genetic variation of past Red Wolf lineages (e.g. ghost ancestry). We analyzed 54,439 loci genotyped across the genome of 413 North American canids and investigated the role that assortative mating with respect to ancestry proportions played in the retention of endangered genetic variation. We report high correlations of inter-chromosomal ancestry proportions that varied with geographic location along Texas and Louisiana Gulf Coast populations, with the stronger signatures reported in the latter. We found that models of assortative mating promoted greater ancestry variance compared with random mating leading to increased efficiency of selection for Red Wolf and ghost alleles. Despite the Red Wolf being extinct in the wild, original, and ghost genomic variation persists in Gulf Coast admixed canids. We suggest two conservation strategies that value and preserve this unique and endangered genomic variation through designed breeding programs. Ultimately the incorporation of this ghost genetic variation would be valuable to boost the genetic viability of the *ex situ* Red Wolf breeding program, create *in situ* redundancy, and avoid extinction for this endemic American wolf species.

## Graphical Abstract

**Graphical Abstract:**
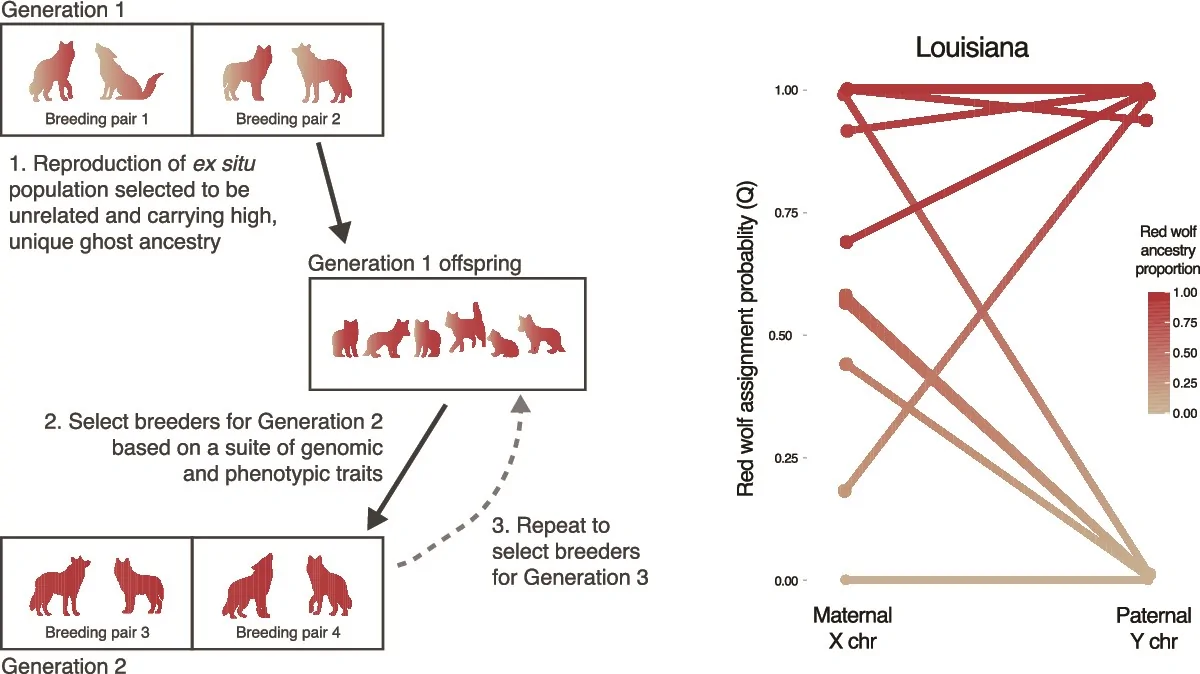

## Introduction

The Red Wolf (*Canis rufus*) is one of the world’s most endangered canids and is endemic to North America. Although the Red Wolf once ranged across most of the eastern United States ecosystems represented by three subspecies (*C. rufus rufus*, *gregoryi*, and *floridanus*), the *ex situ* population that all modern Red Wolves are descendants of was founded by only a single lineage, *C. rufus gregoryi* (USFWS 1990; Waddell and Long 2015). Contemporarily the near entirety of the species is represented by approximately 280 individuals in the *ex situ* breeding program (USFWS 1990; 2022), with approximately 20 to 30 individuals persisting in a reintroduced and heavily managed wild population in northeast North Carolina (NENC) (Murray et al. 2015; Waples et al. 2018; NAS 2020). Paradoxically, despite the Red Wolf being declared extinct in the wild in 1980 after the founding of the *ex situ* population (USFWS 1990), several published studies have declared finding non-negligible amounts of Red Wolf genomic ancestry persisting in coyote populations of the American southeast (Heppenheimer et al. 2018, 2020; Murphy et al. 2019; vonHoldt et al. 2022a, 2022b). What are the evolutionary or ecological mechanisms that support decades of persisting Red Wolf genomic ancestry? And how can these rediscovered admixed genomes carrying lost genomic variation be intentionally leveraged to restore extinct genetic diversity for the future of Red Wolf conservation?

### The recent extinction in the wild of the endemic Red Wolf

Throughout the 20th century, habitat loss and anthropogenic-caused mortality resulted in the species’ precipitous decline (Goldman 1937; Paradiso and Nowak 1971, 1972; Nowak 1974, 2002). By the 1960s, only small, isolated populations of Red Wolves (*C. rufus gregoryi*) persisted on protected private or refuge land in southwest Louisiana (SWLA) and east Texas (TX) (Riley and McBride 1973). Further, many were concerned about the impending threats of hybridization as coyotes (*Canis latrans*) range expanded to fill the ecological niche left vacant by the Red Wolf (Carley 1979; USFWS 1990; Nowak et al. 1995; Fredrickson and Hedrick 2006). Combined with spreading disease, the United States Fish and Wildlife Service (USFWS) removed the last wild Red Wolves from SWLA and east Texas to establish an *ex situ* breeding program. Through this population founding, the species bottlenecked to 12 founders that are genetically represented among all extant Red Wolves. The *ex situ* program supports Red Wolf reintroduced to NENC initiated in 1987. The species is inbred with very low diversity preserved (Fredrickson et al. 2007; Hedrick and Fredrickson 2007; Chambers et al. 2012; Hinton et al. 2013; Brzeski et al. 2014) and recent population viability analyses suggest inbreeding has contributed to reduced litter size (Faust et al. 2016). This negative feedback loop of decaying genetic diversity and fitness will continue to challenge Red Wolf recovery and restoration to their historical range. This may also, in part, be undercut by the presumed lack of public knowledge with roughly 58% of people aware that the Red Wolf is endangered and 32% of those knowledgeable about its recovery program (Lash and Black 2005).

Two independent research groups simultaneously announced their discovery of Red Wolf genetic ancestry in coyotes sampled in SWLA and east Texas (Heppenheimer et al. 2018; Murphy et al. 2019). These individuals represent a unique, historically admixed population (hereafter canids [*C. latrans* × *rufus*]) that encompasses lost, ghost, or extinct genetic ancestry of Red Wolf lineages that are not represented in the present-day *ex situ* breeding program. The extent of Red Wolf genomic ancestry has continued to be identified and quantified in these canids along the American Gulf Coast that carry the highest and oldest Red Wolf genetic information on record (Heppenheimer et al. 2020; vonHoldt et al. 2022a). There is suspected ecological impact of Red Wolf ancestry in these canids, as canids with higher Red Wolf ancestry are larger in body size, a trait that is predictive of their functional role in the local ecology (Hinton et al. 2018; Ward et al. 2018; vonHoldt et al. 2022b). Further, present-day SWLA canids have high amounts of private allelic variation, which likely represent pre-extinction Red Wolf genomic variation (Heppenheimer et al. 2020; vonHoldt et al. 2022a, 2022b). If these animals could serve as new founder lineages of the historic Red Wolf, this could both bolster the genetic health of extant Red Wolves and recover unique, extinct Red Wolf genetics, which is unrepresented in the *ex situ* population. This is now possible given these recent discoveries of ghost Red Wolf genetics that persist in wild canids of SWLA and east Texas (Heppenheimer et al. 2018, 2020; Barnes et al. 2022; vonHoldt et al. 2022a, 2022b).

### The value of hybridization and admixture

Gene flow rapidly moves genomic material, and thus linked traits, between reproductively compatible lineages, resulting in admixed genomes and possibly introgressed signatures when there are fitness consequences (Carlson et al. 2014). Genomic studies increasingly reveal that introgressive exchanges are widespread across taxa and can contribute to ecological novelty, adaptive capacity, and, in some cases, lineage persistence in rapidly changing environments (Supple and Shapiro 2018; vonHoldt et al. 2018; Taylor and Larson 2019; Brauer et al. 2023). The resulting recombinant and admixed genomes may harbor beneficial alleles unavailable through standing variation or mutation alone, admixture has become an important consideration in conservation genetics, especially for small or inbred populations with limited evolutionary potential (Aitken et al. 2008; Hamilton & Miller 2016).

The mechanisms that retain introgressed lineages on the landscape are not well understood. In systems where endangered or declining species hybridize with more common congeners, introgressed ancestry may persist, erode, or increase depending on how drift, gene flow, selection, and mating behavior interact. It is essential to understand these mechanisms, particularly when conservation programs utilize genetic rescue, back-breeding, or de-introgression options to restore lost genomic diversity (e.g. Florida panther, *Puma concolor coryi*; Norfolk Island Boobook owl, *Ninox novaeseelandiae undulate*) (Anderson 1949; Anderson and Stebbins 1954; Harrison 1993; Rieseberg and Wendel 1993; Arnold 1997; Dowling and Secor 1997; Rieseberg et al. 1999; Benson et al. 2011; Crispo et al. 2011; Garnett et al. 2011; Hedrick 2013; Hostetler et al. 2013; Rius and Darling 2014). Of particular relevance to admixed genomes is a de-introgression breeding program, which leverages admixed ancestry derived from an endangered or extinct lineage. Through controlled breeding, genomic screening, and selection, the program progressively increases the representation of ancestry from the endangered or extinct species while reducing ancestry from off-target lineages. Over multiple generations, this approach can reconstruct much of the original genomic composition and diversity of the endangered species, despite its prior decline or extinction in the wild (Amador et al. 2012, 2013, 2014; Shapiro 2017; Quinzin et al. 2019; vonHoldt et al. 2022a; Lawson et al. 2025). Combined with ecological or behavioral mechanisms that already retain introgressed lineages in natural populations, new conservation options emerge that would bolster the genomic diversity of imperiled species and build hope for a species when conventional methods have failed to restore and recovery (Baskett and Gomulkiewicz 2011; Amador et al. 2012, 2013; Vander Wal et al. 2013; Bohling 2016).

### Leveraging assortative mating

Practically, de-introgression programs can utilize a species’ reproductive strategies in a back-breeding program (Shapiro 2017). The goal of a back-breeding program is to reconstitute the original genomic composition through strict control of reproduction with individuals carrying the highly valued and targeted genomic ancestry. This strategy has been applied to resurrect the aurochs (*Bos primigenius*), an extinct bovid species that is assumed to be the ancestor of present-day domestic cattle (Loftus et al. 1994; Edwards et al. 2007; Stokstad 2015).

However, if a species assortatively mates, an individual selects a mate that has a similar trait, this should produce a detectable genomic signature; for humans, this has been characterized as increased genetic variation and population stratification (Yengo et al. 2018). Loci associated with traits selected by mates should show signatures similar to positive, directional selection of increased homozygosity (Risch et al. 2009; Sebro et al. 2010, 2017). Yet, these signatures can be confounded by other genetic correlates and aspects of social structure like reproductive suppression, inbreeding, or gene flow (Astle and Balding 2009).

If ancestry is linked to specific traits that are selected through mate choice, both the causative loci and linked ancestry markers should carry the same signature, with differences observed between autosomes and sex chromosomes (Sampson et al. 2011; Robinson et al. 2015; Sebro et al. 2017; Goldberg et al. 2020). Thus, if assortative mating favors traits derived from the extinct lineage that appear in admixed individuals, these genomic signatures provide a clear empirical window into how ancestry from an extinct species can persist despite its apparent paradox. While inbreeding or trait-based selection reduces genomic diversity at linked loci and thus the effective population size (Ne), admixture and gene flow will counteract this effect by increasing relevant metric estimates (Araki et al. 2007; Reeves et al. 2025). For instance, ancestry-based assortative mating in humans has produced genomic signatures that, when analyzed, highlight the phenotypic cues (e.g. anthropometric, neurological, and immunity) used for reproductive decisions (Norris et al. 2019). Across individuals of known relationships, assortative mating is revealed as bundles of co-ancestry, ultimately leading to genetic isolation between the diverging groups due to mate choice (Muralidhar et al. 2022). Thus, mixed ancestry populations provide an incredible opportunity for population genomic tools to study the impact of mate choice on chromosomal and trait patterns.

Here, we hypothesized that assortative mating with respect to Red Wolf ancestry explains the persisting levels of said ancestry in Gulf Coast canids. We investigated this through empirical and simulated patterns of Red Wolf ancestry of autosomes and sex chromosomes. To connect this with an actionable conservation back-breeding program, we used models to describe the potential impact of selecting breeders with high Red Wolf content. This analysis will demonstrate the transformative role of admixed genomes in endangered species conservation while simultaneously highlighting a new path forward in Red Wolf recovery efforts.

## Methods

### Sample collection

Since 2018, we collected 124 canid blood and tissue samples used for genomic analyses using multiple methods from east Texas and Louisiana, United States, referred to as our focal population. We previously reported high estimates of ghost Red Wolf genomic ancestry in this focal population (Heppenheimer et al. 2020; vonHoldt et al. 2022a, 2022b). We sourced tissue samples from east Texas from roadkill and routine management activities conducted by staff from the Galveston Humane Society and Animal Services on Galveston Island. We obtained blood or tissue samples from an additional 15 coyotes (*C. latrans*) and 94 gray wolves (*Canis lupus*) provided by collaborating agencies and research partners that served as reference samples to infer ancestry and genomic variation of the focal canids (Table S1).

### Genomic DNA and preparation of libraries

We obtained genomic DNA using DNeasy blood and tissue extraction kits (Qiagen) following the manufacturer’s protocol. We quantified DNA on a Fluorometer Qubit system. We prepared the genomic DNA for restriction-site associated DNA sequencing capture (bestRADseq) following Ali et al. (2015). Briefly, we digested genomic DNA with the *SbfI* restriction enzyme followed by ligation of a unique 8-bp barcoded biotinylated adapter that allowed pooling of equimolar amounts of 48 DNA samples. We used a Covaris LE220 to randomly shear the pools to approximately 400 nucleotides in average length fragments that were enriched for the adapter using a Dynabeads M-280 streptavidin binding assay. These enriched pools were prepared for Illumina NovaSeq 6000 paired-end (2 × 150 nt) sequencing at Princeton University’s Lewis-Sigler Genomics Institute core facility using the NEBnext Ultra II DNA Library Prep Kit. We used Agencourt AMPure XP magnetic beads for library cleaning and size selection of fragments that were 300 to 400 nucleotides in size.

Reduced-representation genomics approaches (such as bestRADseq) remain powerful and cost-effective tools for population genetic analyses by sampling thousands of loci across the genome for hundreds of individuals at a fraction of the cost of whole-genome sequencing. The method we use (bestRADseq) is especially valuable for large sample sizes where breadth of population coverage is essential but also faces the same universal limitations of locus dropout or allele bias, especially when only a fraction of the genome is sequenced. Despite these typical limitations, reduced-representation methods provide a reliable and efficient tool for characterizing population genomic variation and demography.

### Processing and cleaning the sequence reads

We retained sequence read pairs that contained the unique barcode and remnant *SbfI* recognition site in *STACKS* v2.6 (Catchen et al. 2013; Rochette et al. 2019). We first rescued our barcoded reads in the *process_radtags* module (up to two nucleotide mismatches permitted) and retained reads that had a quality score ≥ 10. We removed PCR duplicates (*clone_filter* module) followed by mapping to the reference dog genome CanFam3.1 assembly (GCA_000002285.2; Lindblad-Toh et al. 2005) using *bwa-mem* (Li 2013a). We also included the Y chromosome (KP081776.1; Li et al. 2013b) with the CanFam3.1 reference assembly. After alignment, we removed reads with a low mapping quality (MAPQ<20) and converted the resulting SAM files to BAM format (*Samtools* v0.1.18, Li et al. 2009).

### Catalog construction and SNP variant genotyping

Along with our newly collected data, we included additional bestRADseq data previously published from 45 focal canids and 107 additional North American reference canids (Table S1). This provided a full coverage of all ancestries possible for the 197 focal canids that was partitioned among North American canids: coyote (*n* = 93), gray wolf (*n* = 94), eastern wolf (*Ceraphron lycaon*, *n* = 10), *ex situ* Red Wolf (*C. rufus*, *n* = 10), and domestic dog (*Canis lupus familiaris*, *n* = 9). We constructed a catalog of all polymorphic sites possible for newly collected and previously published data. We implemented the *gstacks* and *populations* modules in *STACKS* v2.6 (--vt-alpha,--gt-alpha, *P* = 0.01) to confidently discover and annotate polymorphic sites. We then used *VCFtools* v0.1.17 (Danecek et al. 2011) to exclude singleton and private doubleton alleles, remove loci with more than 90% missing data across all samples, and remove individuals with more than 20% missing data. We filtered to exclude sites with a minor allele frequency (MAF < 0.03) and allowed up to 80% genotyping rate per locus (--*geno* 0.2) in *PLINK* v1.90b3i (Chang et al. 2015). For demographic analyses, we constructed a statistically neutral and unlinked dataset of SNPs by excluding sites within 50-SNP windows that exceeded genotype correlations of *r* = 0.5 (--*indep*-*pairwise* 50 5 0.5; a proxy for linkage disequilibrium or LD) and SNPs that significantly deviated from Hardy–Weinberg Equilibrium with the argument --*hwe* 0.001.

### Sample assessment and clustering as proxies for global ancestry proportions

We conducted both a nonmodel and model-based clustering analysis on the statistically neutral and unlinked set of SNP loci to assess patterns of genetic clustering of samples and further inform our hypothesis for downstream ancestry assessment. We conducted a principal component analysis (PCA) in *FlashPCA v2.1* (Abraham et al. 2017) as a nonmodel clustering method. We used a supervised maximum likelihood framework with *Admixture* (Alexander et al. 2009) to assess posterior probability assignments separately at three partitions (*K*) for the reference groups gray wolf, coyote, and Red Wolf. We utilized these probabilities as a proxy for global genomic ancestry proportions.

### Private and ghost alleles

We utilized the *populations* module in *STACKS* v2 to identify the alleles private to each canid group on the minimally filtered SNP set. We conducted a rarefaction method for private allele richness per locus while controlling for sample size variation in the number of genomes sampled in the program *ADZE* (Szpiech et al. 2008) with the parameter G of sample size set to 100. To tabulate the number of private alleles carried by each individual, we first removed loci that contained any missing data in our subset of SWLA focal canids from two parishes in Louisiana (Cameron and Jefferson Davis; Table S1). We also identified which alleles were exclusive to the *ex situ* reference Red Wolves after further restricting the analysis to loci with high loadings on a principal component that were diagnostic for Red Wolves (Zou et al. 2010). Finally, we selected loci that had PCA loadings and F_ST_ estimates in the 5th and 95th percentile tails (*PLINK*’s --fst function that implements Wright’s F_ST_; Weir and Cockerham 1984).

### Relatedness and effective population size

We estimated relatedness within each of the focal populations of Galveston Island and SWLA. In SWLA, we used paired global positioning system coordinates for 23 Cameron Parish canids to explore relationships among three field sites: the FR Ranch Preserve at Moore-Odom (FR, *n* = 12), Apache Corp private land (*n* = 10), and Cameron Prairie National Wildlife Refuge (NWR, *n* = 1). We filtered the statistically neutral and unlinked SNP set to retain loci with a high minor allele frequency (--*maf* 0.3) and pruned with a more stringent genotypic correlation threshold (--*indep*-*pairwise* 50 5 0.2) (Huisman 2017). We used the R package (R Core Team 2021) *related* to calculate relatedness coefficients between each pair of canids (Pew et al. 2015) and the *coancestry* function for the dyadic likelihood estimator (dyadml = 1; Milligan 2003) that allowed for any potential of inbreeding events (allow.inbreeding = TRUE).

To understand the underlying degree to which SWLA canids may show social structuring as reflected by lower *N_e_* (vonHoldt et al. 2024, 2025), we estimated *N_e_* for *ex situ* Red Wolves, canids from SWLA, and reference coyotes from western United States (California, Idaho, Nevada, Washington, and Wyoming). We conducted this for the canids at our SWLA study site (not eastern Texas) given the landscape is not densely populated and we previously collared these animals (vonHoldt et al. 2022b), compared with tissue samples acquired from carcasses through roadkill or other sources stated in the Methods. We focused on estimates from the past 200 generations, which were presumed to be more accurate than deeper history. We implemented *GONE* (Santiago et al. 2020), an LD-based method that accounts for drift and thought to be more accurate than coalescence estimators. We limited our estimates to autosomal SNP data and translated generations into years using an average of 3 yr per generation as the unit of time (vonHoldt et al. 2008; Mech et al. 2016).

### Assortative mating

To explore a potential signature of assortative mating, we focused on the chromosome-wide ancestries on the X and Y sex chromosomes. We considered positive evidence of assortative mating as a positive inter-sex chromosome correlation of Red Wolf ancestry, as quantified by supervised clustering, in male canids. We first bioinformatically inferred sex for each animal. For male inferences, we counted the number of reads that mapped to the canine Y chromosome. We did not filter loci on the sex chromosomes for linkage or deviations from neutrality. We then quantified the probability of Red Wolf ancestry for each of the sex chromosomes for the bioinformatically inferred males. Further, we focused on male canids from Galveston Island and Cameron Parish (Louisiana) as these two regions have the highest relative density of samples while representing landscapes with different rates of human-caused mortality (Barnes et al. 2022; vonHoldt et al. 2022b). We estimated observed heterozygosity (H_O_) in *PLINK* using the statistically neutral and unlinked SNP set found on the autosomes, which represented a total genome length of 2,202,059,258 nucleotides.

We next separately phased the autosomes, and each of the sex chromosomes separately for all canids and indicated sex as it was inferred from the Y chromosome methods described above. We phased using *SHAPEIT* v2.r790 (Delaneau et al. 2012). For the X chromosome, we included the *--chrX* flag to impute missing data for males and phase the X chromosome of females; for the Y chromosome, we treated it as diploid given it complements the X chromosome in the pseudoautosomal region (first 6.7 Mb of the X chromosome; Raudsepp et al. 2012).

### Modeling effects of assortative mating and selection

We developed an individual-based model to explore Red Wolf ancestry outcomes under two mate choice scenarios, random mating and assortative mate choice, and two selection pressure conditions, either no selective advantage for Red Wolf ancestry or strong positive selection for Red Wolf ancestry as might result from the selection of breeders in a breeding program. We represented each individual by their multi-locus diploid genotype drawn from a selection of 2,447 autosomal loci (Table S2). We selected loci to include loci that carried identified ghost alleles, Red Wolf-specific ancestry information, and loci not informative with respect to ghost or Red Wolf variation (referred to as background loci). The ghost and Red Wolf-specific alleles were combined to represent total Red Wolf ancestry while the background loci acted as a control group not associated with ancestry. The ancestry-specific loci were selected to have a minimum depth of 10× sequence coverage. We initialized the model with the first generation being drawn from the empirical observed allele distributions.

We modeled female mate choice using the method developed by Fredrickson and Hedrick (2005), which they applied to Red Wolf and coyote interbreeding. To establish a total lack of mate choice, we randomly assigned a male to each female for reproduction. To compare the effects of various strengths of positive size-based assortative mating, we modeled mate choice by matching each female to a male with an equivalent (or most comparable) score of Red Wolf ancestry from a pool of potential mates. We were guided by the previous finding that size (i.e. mass) significantly co-varies with genome-wide Red Wolf ancestry proportion (Pfeffer et al. 2022; vonHoldt et al. 2022b), likely because body size is a highly polygenic trait. Size can be expected to act as an indicator of ancestry and lead indirectly to ancestry-based assortative mating (Norris et al. 2019). The pool of potential male mates from which each female was allowed to choose represented the strength of the mate choice, with a larger pool permitting the female a simulated environment of increased scrutiny and thus a closer match. We considered a range of *n* = 1 to 5 individuals in the pool of potential male mate options. We represented monogamy by allowing females to sequentially choose mates from the pool of unpaired males and we limited each individual to a maximum of membership to a single breeding pair.

We modeled the population through a series of nonoverlapping generations, where 1,000 individuals from each generation were selected to act as breeders in the subsequent generation. We modeled reproduction through the creation of litters of four to seven offspring by each breeding pair (Kilgo et al. 2017). As the selected loci were previously filtered to be unlinked, we assumed independent segregating loci that allowed each locus to be inherited at random from either parental chromosome. Offspring received a random set of alleles from each parent to produce their diploid genome. To model neutral evolution, we randomly selected survivors. Under the scenario of directional selection in a breeding program, we included the conditional that an individual’s survival probability was directly proportional to their Red Wolf ancestry score. This generated a strong selection pressure in favor of higher levels of Red Wolf ancestry. We repeated this for a total of 10 generations for each mating scenario and each selection condition. To reduce the model run time, each simulation included a randomly selected subset of alleles from a total of 100 ancestry informative and ghost loci and 100 background loci.

We analyzed the distribution of Red Wolf ancestry scores under different mate choice assumptions and selection regimes. We used the number of Red Wolf alleles, encompassing both ghost alleles and Red Wolf-specific ancestry informative alleles, relative to the total number of modeled Red Wolf loci (*n* = 200) as a metric for the Red Wolf ancestry score in our comparisons. This could range from zero where none of the loci displayed Red Wolf-associated alleles and one where all relevant loci contained Red Wolf-associated alleles. To assess changes in genetic variation and test for divergence across the simulated generations and between simulation conditions, we used graphical representations to track the distribution of Red Wolf ancestry scores over time in each simulated population. We recorded the mean and the interquartile range of ancestry scores in the population at each generation under each set of conditions. We repeated each scenario 500 times and recorded the 95% range of model outputs of the summary statistics in each case. We compared the results for each simulated population with the other simulated populations and to other generations within the same simulation run.

## Results

We discovered 642,263 loci across 413 canids with a mean insert length of 357.2 bp (s.d. = 105.2) and average per-sample sequence depth of 12.4× (s.d. = 7.0, min = 1.4, max = 54.0). We excluded 33 canids that had more than 20% of the genotype data missing (Table S1). After filtering for MAF < 0.03 and missingness, we obtained 54 439 variants genotyped in 380 canids; this included 197 focal samples, where 47 were from Galveston Island and 74 from SWLA (Cameron Parish = 44). Further filtering retained 34,765 statistically neutral and unlinked SNP loci.

### Gulf Coast canids are most genetically similar to Red Wolves

**Fig. 1:**
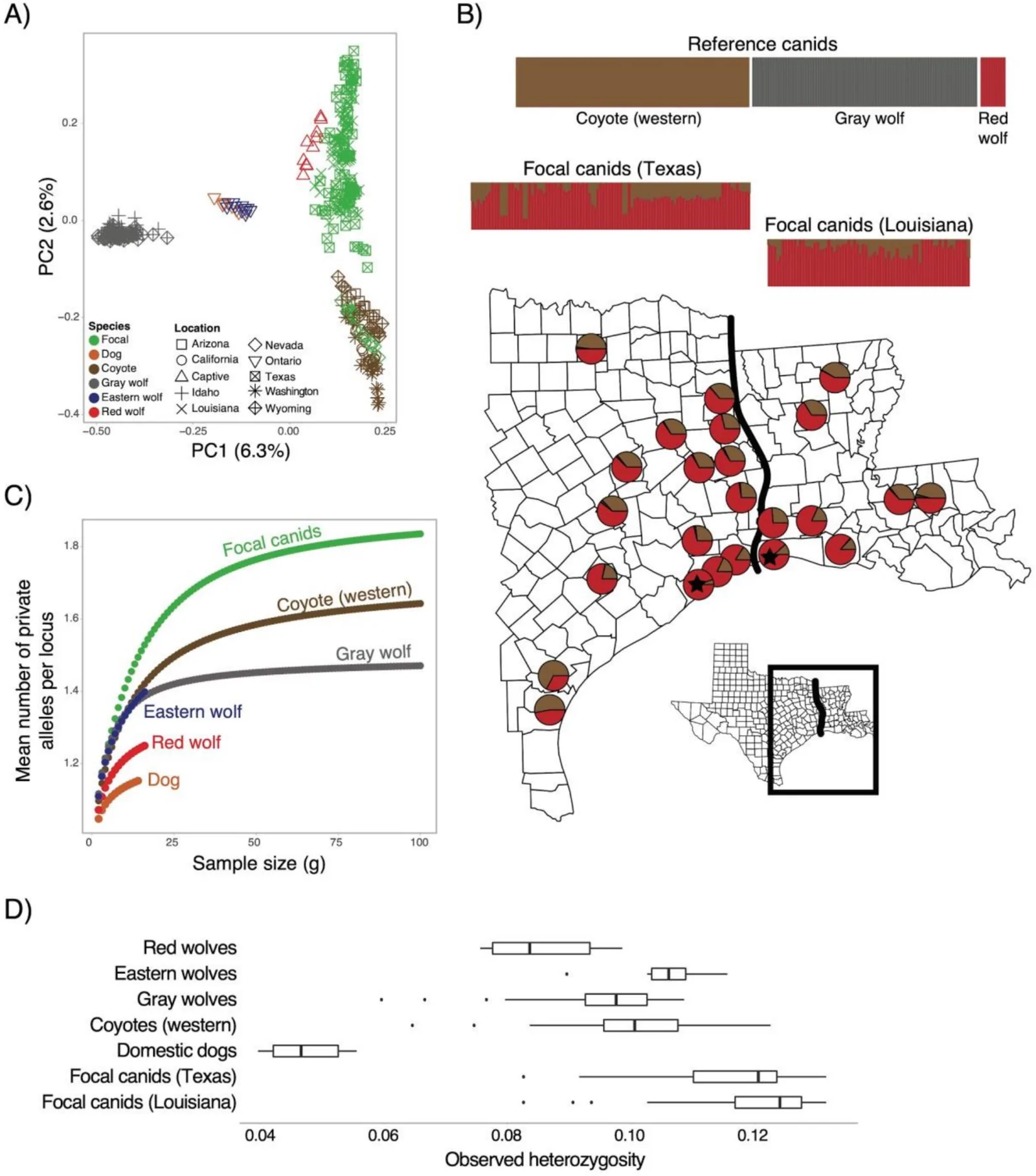
Genetic variation and structuring of 169 focal canids of southwestern Louisiana and Texas relative to reference canid lineages of North America genotyped at 34765 statistically neutral and unlinked SNP loci in the (A) PCA, (B) supervised structure with averages per county or parish on their geographic origins (stars indicate the target county of Galveston and parish of Cameron), and (C) a rarefaction analysis. (D) Boxplots of observed heterozygosity for each focal population and the five references.

Focal canids from east TX and SWLA did not cluster with dogs or eastern wolves (Fig. 1A), thus we excluded those two lineages as possible references for all downstream analyses. Under supervised clustering to three reference groups (gray wolf, coyote, and Red Wolf), we found that the focal canids of TX and SWLA predominantly were assigned to Red Wolves (mean *Q* = 0.798), followed by western coyotes (*Q* = 0.197) and gray wolf (*Q* = 0.005) (Fig. 1B; Table S1). Thus, we further excluded gray wolf as a possible genetic ancestry reference canid.

We tabulated the number of alleles private to each canid group out of 54,439 SNP loci and found that dogs, eastern wolves, and *ex situ* Red Wolves lack any private alleles (Table 1). Gray wolves carried the most private alleles (*n* = 2,502), followed next by the focal canids (*n* = 1,039), and then western coyotes (*n* = 162). A rarefaction analysis confirmed this pattern after controlling for sample size, whereas the focal canids from TX and SWLA carried the highest mean number of private alleles per locus (Fig. 1C). Both focal populations were more heterozygous for statistically neutral and unlinked loci than the reference lineages (mean H_O_, Galveston = 0.12, and Cameron Parish = 0.12) (Fig. 1D).

### Low genetic relatedness and high effective size estimates

**Fig. 2:**
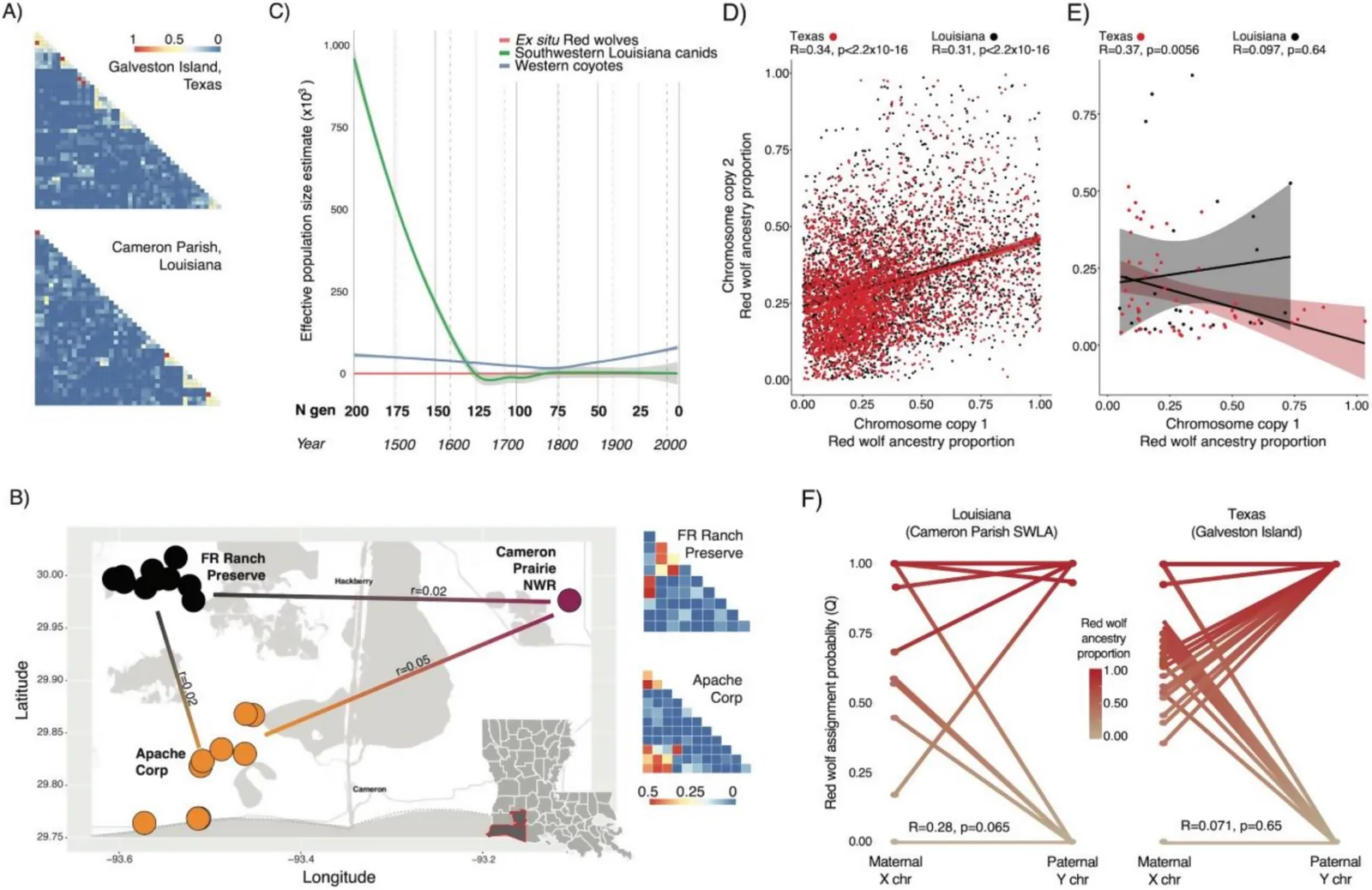
(A) Heatmap of pairwise relatedness estimates of canids in two focal populations. Scale bar depicts relatedness. (B) Within Cameron Parish, canids with known sampled locations (circles) and the average inter-location relatedness depicted by lines between the three study sites and the relatedness (r) values on lines. Heat maps of intra-location relatedness values for two locations are proved to the right. (C) Effective population size estimates for 10 *ex situ* red wolves, 71 southwestern Louisiana canids, and 93 western coyotes. The *x* axis depicts time units, the number of generations back (N gen; solid vertical lines) and year (dashed vertical lines) as per 3 yr/generation and a loess smoothing function for each temporal estimate. Comparison of Red Wolf ancestry proportions with regression correlations across phased copies of (D) all the autosomes and (E) two X chromosomes for only females. (F) Assessment of supervised ancestry assignment probability for the maternal X and paternal Y chromosomes (males only) from two focal populations. The lines connect the two sex chromosomes for each individual analyzed and color gradient indicates the ancestry probability for each chromosome. Regression correlation coefficients and *P*-values are indicated.

We increased the filtering stringency to retain 104 SNPs for which we estimated pairwise relatedness within each of the focal populations. We found a low level of average inter-individual relatedness of *r* = 0.06 in Galveston (s.d. = 0.12) and Cameron Parish (s.d. = 0.11), with only a few individuals showing higher levels of relatedness (*r* > 0.4) (Fig. 2A). When we compared relationships among the three different Cameron Parish field sites, we found intra-group relatedness estimates (*r*_FR_ = 0.12, *r*_Apache_ = 0.10) were higher than inter-group comparisons (*r*_FR-NWR_ = 0.02, *r*_Apache-NWR_ = 0.05, *r*_FR-Apache_ = 0.02) (Fig. 2B). Of the 56 pairwise relatedness comparisons at the FR Ranch Preserve, 11 pairs had *r* > 0.25, suggestive of close relatives at the 1st- or 2nd-degree relationship status. Similarly, seven of 45 pairwise estimates in Apache Corp had similarly high relatedness (*r* > 0.25). No inter-group relatedness estimates were high (*r* > 0.25), suggestive of recent or effective genetic connectivity.

**Table 1:** Nucleotide diversity (*π*), the number of polymorphic sites, and the number of private alleles per canid lineage out of 54 439 SNP loci.

Canid lineage	*π*	No. polymorphic sites	No. private alleles
Dog	0.066	10,784	0
*ex situ* Red Wolf	0.117	24,107	0
Eastern wolf	0.086	15,789	0
Gray wolf	0.122	27,425	2,502
Coyote	0.094	36,117	162
Focal canids^a^	0.114	48,926	1,039

a Texas and Louisiana populations that have known Red Wolf admixture

We used 34 465 statistically neutral and unlinked SNP loci to estimate *N_e_* for each demographically distinct canid group (*ex situ* Red Wolves, western coyotes, and southwestern Louisiana canids; Heppenheimer et al. 2018, 2020; vonHoldt et al. 2022a, 2022b). Overall, the historical *N_e_* estimates matched demographic knowledge, where western coyotes showed consistently larger estimates (200- and 5-generation ago *N_e_* = 53,572.1 and 76,534, respectively) than *ex situ* Red Wolves, which remained very low over the past 200 generations (200 and 5 generations ago, *N_e_* = 4.3 and 44.1) (Fig. 1C). The canids in SWLA displayed a rapid decline between 200 and 125 generations, from *N_e_* = 842,888 to 5,761.4 with a subsequently slow decline until present day (5-generations ago *N_e_* = 225.8). Presently (past 15 generations or approximately 45 yr), we found that *ex situ* Red Wolves had an average of *N_e_* = 46.2 (range = 37.8 to 56.1) and western coyotes *N_e_* = 58,572.8 (range = 396.4 to 87,697.3). Canids in SWLA represent an effective population size of 599.2 individuals (range = 192 to 2,375.9).

### Inter-chromosome correlations with genomic ancestry

We found each phased chromosome’s global ancestry proportions of *ex situ* Red Wolf was significantly and positively correlated with its homologous chromosome (*R* = 0.3, *P* < 2.2 × 10^−16^) in both geographic sites (Galveston and SWLA) (Fig. 2D, Fig. S3). We further separately analyzed females and males, after determining their sex bioinformatically. An average of 13,806.6 reads (s.d. = 13,984.5) mapped to the canine Y chromosome and formed a bimodal distribution of reads mapping per individual canid (Table S1, Fig. S2). The first peak accounted for canids with no or very few reads (≤2,802 reads) mapping to the Y chromosome that were inferred as females (*n* = 195). The second peak were canids inferred as males (*n* = 185) with ≥12,973 mapped reads. We found that the diploid X chromosomes of 82 females from the focal populations had geography-specific patterns, with high correlation between the two chromosomes for Louisiana focal canids but decoupled for focal canids of eastern Texas (Fig. 2E). The X chromosomes of SWLA canids were not significantly correlated in their ancestry proportions (*R* = 0.1, *P* = 0.64) while X chromosomes in Texas displayed a significantly higher and negative correlation (*R* = 0.37, *P* = 0.006), where a minority of females carried divergent ancestry proportions for each of their X chromosomes (Fig. S4).

The X-Y chromosome comparisons for male canids in Galveston Island (TX) and Cameron Parish (SWLA) demonstrated the Y chromosomes were not admixed and thus represented only one of two paternal ancestry types (coyotes or Red Wolf). We found a significant positive correlation of Red Wolf ancestry between the sex chromosomes (Pearson’s product–moment, Galveston: *cor* = 0.800, *t* = 10.0, df = 56, *P* = 4.79 × 10^−14^; Cameron Parish: *cor* = 0.586, *t* = 5.3, df = 53, *P* = 2.54 × 10^−6^) when we included reference coyotes and Red Wolves. A linear regression revealed similar trends across geographic sites, with a stronger albeit nonsignificant correlation of Red Wolf ancestry in Louisiana canids (*R* = 0.28) compared with Galveston Island (*R* = 0.071, *P* = 0.65) (Fig. 2F).

### Selection pressure and assortative mating have an interactive effect on Red Wolf ancestry

**Fig. 3:**
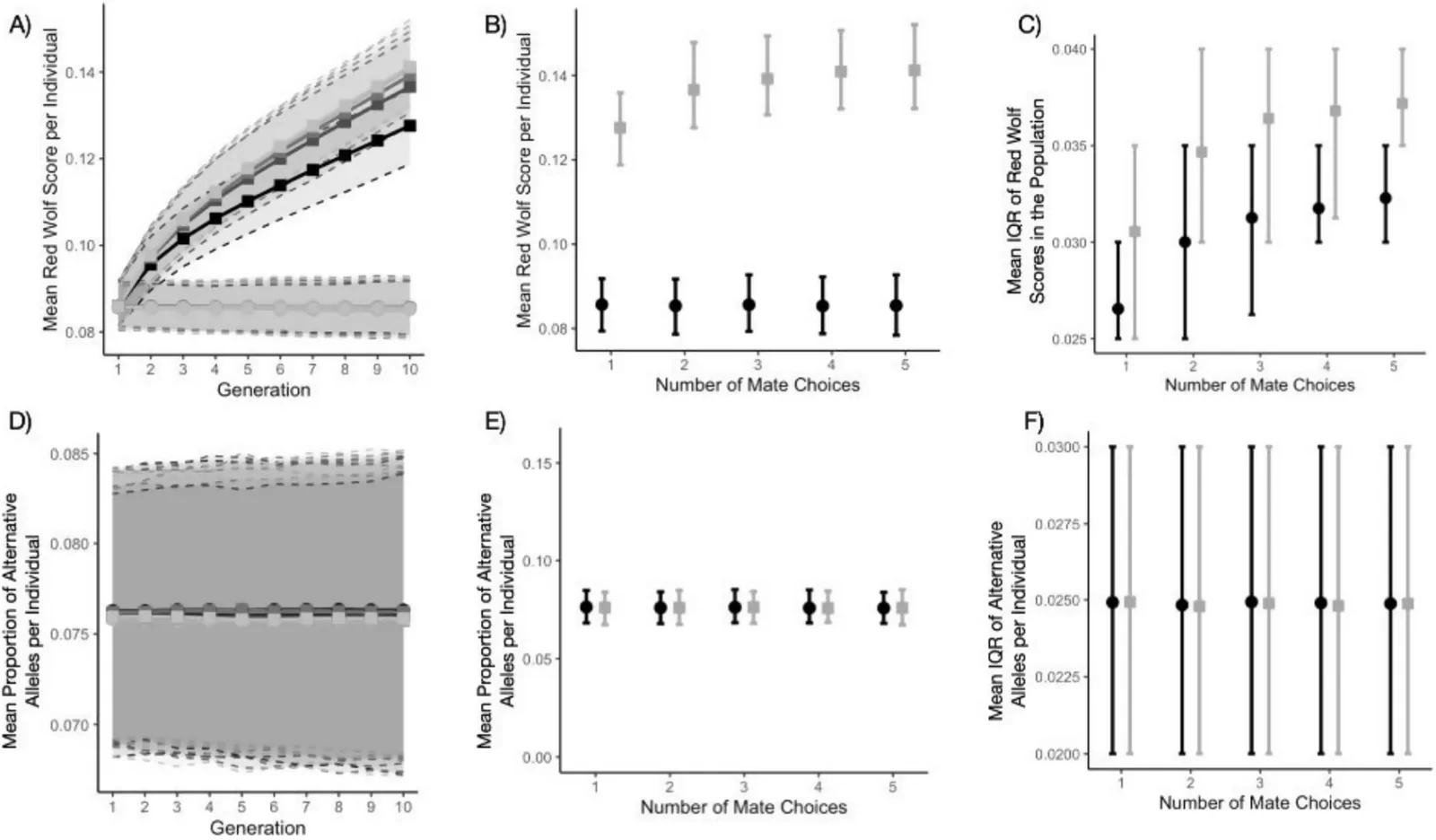
Simulation of Red Wolf ancestry over 15 generations of assortative mating with respect to Red Wolf ancestry (positive selection scenario) and a no selection scenario. (A) The mean proportion of Red Wolf ancestry per individual, and (B) the final mean proportion and (C) interquartile range in the 15th generation. (D) The mean proportion of alternative neutral alleles per individual over 15 generations, and (E) the final mean and (F) interquartile range after 15 generations under 1 to 10 mate options.

We modeled size-assortative mate choice scenarios ranging from random through strong positive assortment, under both neutral and positive selection conditions, and investigated their temporal impact on Red Wolf ancestry scores. We found that size-assortative mate choice led to a slight increase in the range of Red Wolf ancestry scores in the population after 10 generations while positive selection led to a strong increase in the mean Red Wolf ancestry score over time. In the absence of a selective advantage, there was no mean change in Red Wolf ancestry scores across the 10 simulated generations (mean propr. = 0.086) (Fig. 3A and B). In comparison, under the directional positive selection scenario where we simulated a selective advantage for high Red Wolf ancestry, we found that the mean number of Red Wolf genetic markers per simulated individual qualitatively increased linearly over time (mean, t_1_ = 0.086 and t_10_ = 0.137), with different rates of increase in different mate choice scenarios. Under both selection scenarios, increasing the strength of the mating preference resulted in an increased variation of Red Wolf ancestry scores between individuals in the population, although there was high variation between simulation runs (Fig. 3C).

We then permitted an increase in the number of mate choice options per female. We found this had a nonlinear effect on the range and mean of ancestry scores in the population, with a shallower rate of increase of Red Wolf proportions with a greater number of mate choices possible (Fig. 3B and C). Selection pressure and mate choice behavior had an interactive effect on the mean Red Wolf score with the impact of mate choice depending on the presence of selection. The steepest change in the mean and range of Red Wolf ancestry scores was observed between the final generations of the random mating pattern (mean = 0.128, IQR = 0.031) and the weakest assortative pattern of two mate choices (mean = 0.137, IQR = 0.033) in the presence of selection. When we further increased the number of mate options to simulate a stronger preference, the model displayed progressively smaller changes to the population at each incremental increase with identical outcomes in the last two mate choice conditions (mean ghost propr. for mate choice, four options = 0.141, range = 0.037; five options = 0.141, range = 0.037) (Fig. 3B). Neither selection nor mate preference influenced the distribution of neutral alleles in the population. The mean number of alternative neutral alleles per individual remained constant (mean ~ 0.75) over the 10 generations for both selection scenarios and all mating behaviors (Fig. 3D and E). The interquartile range of alternative neutral alleles after 10 generations is similar under all mate choice scenarios (IQR = 0.025) (Fig. 3F).

## Discussion

While the Red Wolf was declared extinct in the wild in 1980, our goals were to uncover the possible mechanisms by which Red Wolf genomic ancestry has persisted for decades in the unique canids of the American Gulf Coast (Murphy et al. 2019; Heppenheimer et al. 2020; Barnes et al. 2022; vonHoldt et al. 2022a, 2022b). These canids likely represent historical, pre-extinction admixture with the Red Wolf during their precipitous decline in the mid-century due to eradication programs, supported by our report that local effective population size increased in the canids of southeastern Louisiana over the past 25 generations (~75 to 100 yr before present, Fig. 2C). As these introgressed canids persisted and continued to carry, perhaps amplify, pre-extinction ghost genetic variation, the mechanisms that retain introgressed lineages on the landscape are not well understood.

Here, we explored the possibility that one mechanism for the retention of ghost and canonical Red Wolf ancestry could be explained through assortative mating based on unsampled phenotypic traits that are likely shaped by wolf-like genomic ancestry (vonHoldt et al. 2022b). Compared with simulations of random mating, mating preferences anchored on ancestry proportions revealed a trend of maintaining a broader range of ghost ancestry (Fig. 3). Modeling assortative mating in the southwestern Louisiana landscapes sampled, where human-caused mortality appears relatively lower, is easily a realistic scenario (vonHoldt et al. 2022b). Given larger canid body size is associated with higher levels of ghost genetic variation, size-assortative mating could play a role in maintaining or even amplifying ghost ancestry in a wild population of introgressed canids. Introgression contributes not only novel genetic variation and adaptive capacity but also supports the continuation of an extinct genomic lineage, such is the likely case of the Red Wolf. Further, individuals that carry substantial amounts of introgressed extinct genomic variation represent a bold and unconventional tool for species conservation and genetic rescue, particularly for critically endangered species (Baskett and Gomulkiewicz 2011; Amador et al. 2012, 2013; Vander Wal et al. 2013; Bohling 2016; Bohling et al. 2016). De-introgression conservation programs typically aim to leverage natural reproduction to transfer and concentrate genomic ancestry fragments in as few of unrelated individuals as possible to rebuild the genomic composition and diversity that existed pre-extinction (Shapiro 2017), offering a pathway for restoring lost lineages.

While we corroborate previous research that American Gulf Coast canids carry substantial levels of ghost and *ex situ* Red Wolf genomic ancestry (Murphy et al. 2019; Heppenheimer et al. 2020; vonHoldt et al. 2022a, 2022b), we further investigate chromosomal patterns for signatures of mating with respect to ghost genetic variation. We found that patterns of Red Wolf ancestry blocks were highly correlated between autosomes, a possible indication of assortative mating (Seehausen et al. 2014) albeit with little to no reproductive barriers between red wolves and coyotes (Hinton et al. 2018), with the strongest assortment found in Louisiana canids, a region previously shown to carry the oldest admixture events between coyotes and Red Wolves (vonHoldt et al. 2022b). Such a contact zone and geographical assortment is valuable for exploring the fate of admixed genotypes and phenotypes (Barton & Hewitt 1985; Mallet 2005; Uy et al. 2018; Edelman and Mallet 2021), as well as preserving the reservoir of the Red Wolf’s original genomic composition.

### Innovating a role of back-breeding and de-introgression for endangered species conservation

**Fig. 4:**
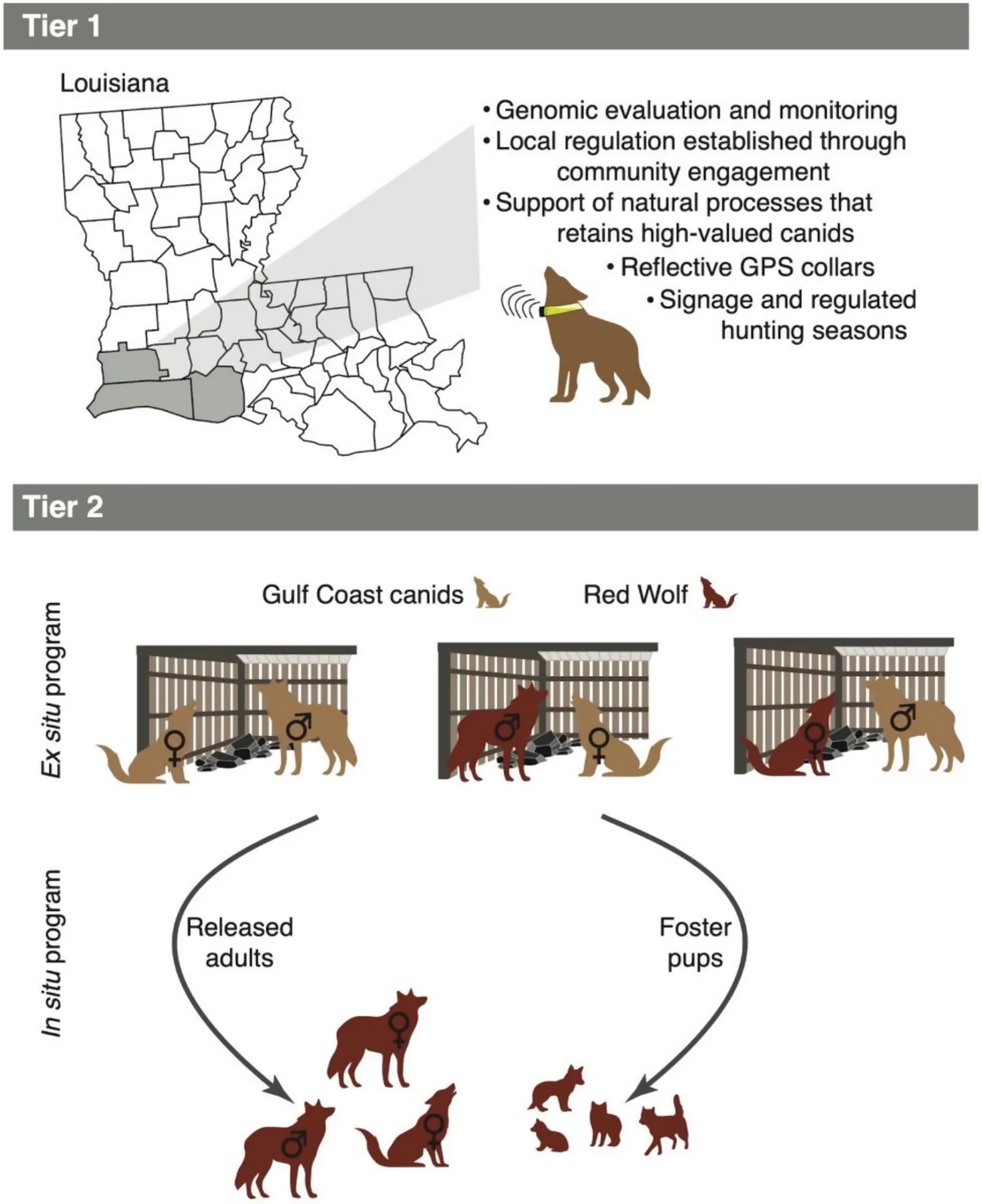
Proposed design for preserving (Tier 1) *in situ* ghost and Red Wolf genetic variation paired with a de-introgression back-breeding design (Tier 2) to restore the genomic diversity of the original Red Wolf.

As wild canid populations that carry substantial amounts of ghost and Red Wolf ancestry have persisted without federal regulatory protection, we propose a two-tiered unconventional design to exhibit the immense value of these admixed genomes for rescuing the genetically depauperate and critically endangered Red Wolf, that outside of the heavily managed reintroduced NENC population, is extinct in the wild (Fig. 4). The first tier is focused on *in situ*, conventional conservation efforts. Private and federal land offers up valuable opportunities to preserve natural evolutionary and life-history processes without a human component other than adjusted species regulations. A focus on regional, not state-wide, policies, utilizing clear engagement with local communities that buy-in to reduced human-caused mortality on their managed landscapes, would boost the long-term stability of ghost and Red Wolf genomic variation. Monitoring programs that implement signage, reflective GPS collars, and easy reporting mechanisms would provide immediate support for a regional population deemed valuable in terms of Red Wolf genomic heritage and composition. The second tier is an *ex situ* back-breeding program design aimed to concentrate ghost genomic variation and rebuild the pre-extinction Red Wolf genomic diversity as a resource for the *ex situ* Red Wolf breeding program and species survival.

### Back-breeding design

We propose two approaches for the de-introgression and recovery of ghost Red Wolf ancestry. First, our analyses presented here support the projected outcome that breeding high ghost-content canids will amplify said genomic variation over several generations. Further, the evidence for ancestry-based assortative mating in the Gulf Coast canid population suggests that this amplification will be more efficient than in a population experiencing random mating (Muralidhar et al. 2022). The ancestry variance maintained by natural patterns of assortative mating provides a broader range of ancestry proportions from which high Red Wolf ancestry individuals can be selected for breeding. Although the model assessed assortative mating that would presumably explain the wild population to date, the same principles would be applied in an *ex situ* program for back-breeding which would enhance the strength of assortative mating. Each generation would follow standard operating procedures to minimize relatedness while maximizing Red Wolf ancestry and ghost genetic variation. Overall, we suggest considering three difference cross compositions for *ex situ* breeding experiments: 1) male and female Gulf Coast canid cross, both with high Red Wolf ancestry; 2) high Red Wolf content female from the Gulf Coast crossed with an *ex situ* male Red Wolf; and 3) high Red Wolf content male from the Gulf Coast crossed with an *ex situ* female Red Wolf. The expectation is that when any *ex situ* Red Wolves are involved, progeny are expected to always show increased *ex situ* Red Wolf ancestry and linked morphology. We further suggest that within the first *ex situ* breeding experiment, male and female Gulf Coast canids be paired assortatively according to their Red Wolf ancestry content to maximize the ancestry variance in the subsequent generation. Breeding pairs in the second and third scenarios would not be paired by ancestry and would instead serve to reintroduce genetic variance lost to the current Red Wolf population. Second, we explore the impact and outcome of *in situ* attempts at pre-extinction Red Wolf de-introgression. An *in situ* breeding experiment could be accomplished via fostering *ex situ* Red Wolf pups into a SWLA den. With *in situ* efforts, naturally occurring assortative mating in combination with the positive selection pressure created by favoring Red Wolf litters is the expected mechanism by which Red Wolf ancestry (contemporary and ghost) should increase. Such expectations could be simulated with the a priori genomic information of introduced and *in situ* individuals.

### Integrating across unconventional options

These two different breeding experiments provide a dual approach to examining the outcome of back-breeding for species conservation. For instance, *ex situ* breeding provides the ability to control individual breeders and document changes in morphology associated with de-introgression. Detailed morphometric and genomic monitoring becomes more difficult in a wild situation, but *in situ* efforts have the advantage of keeping individuals in their current environment where ecological and behavioral dynamics can be annually assessed. This raises the question: Why do anything at all? If the current focal canids can maintain, perhaps even magnify, Red Wolf ancestry, we propose that these canid populations should be elevated in their conservation value for increasing the genomic diversity and ancestral genome restoration of the current Red Wolf *ex situ* population. The two aforementioned proposed breeding experiments are also not mutually exclusive with such a suggestion. Incorporating activities such as fostering or translocation events would bridge these two strategies and promote increased availability of individuals for assortative mating (Volis and Blecher 2010). A hybrid implementation (inter situ) that integrates aspects of both strategies would build up the genomic diversity and promote the ecological restoration of the Red Wolf-like canids on the landscape of southwestern Louisiana. Such an approach would provide “off-site” low-cost management within a natural landscape and is expected to become a self-maintaining population of the endangered species (Red Wolves, in this case). The admixed canids of the Gulf Coast have substantial conservation value for the Red Wolf as they retain high amounts of contemporary Red Wolf ancestry and unique genetic variation of past Red Wolf lineages.

## Supplementary Material

Supplemental_Tables_esag010

private_alleles_SWLATX_SNP_names_esag010

Supplemental_Figures_esag010

## Data Availability

All sorted BAM files produced in this study were deposited on NCBI SRA under the accession PRJNA1225642 and individual sample accession numbers are provided in Table S1.

## Benefits sharing

Benefits from this research accrue from the sharing of our data and results on public databases as described above. More broadly, our research explores questions about the life histories that may impact the conservation of a closely related endangered species, and we are committed to institutional capacity building for preserving biodiversity. Research findings will be shared with federal and state agencies.
