# Supplementary material for: The paradoxical extinction: Exploring signatures of assortative mating as a possible mechanism that maintains canonical Red Wolf genetic ancestry in the American Gulf Coast canids
Source: J Hered. 2026 Jan 30;117(5):1059–72. doi: 10.1093/jhered/esag010 (PMC13539447; doi:10.1093/jhered/esag010)

Figure S1. Per principal component variation across all focal and reference canid lineages of North America genotyped at 34,765 statistically neutral and unlinked SNP loci. Percent explained by each component is provided in parentheses.

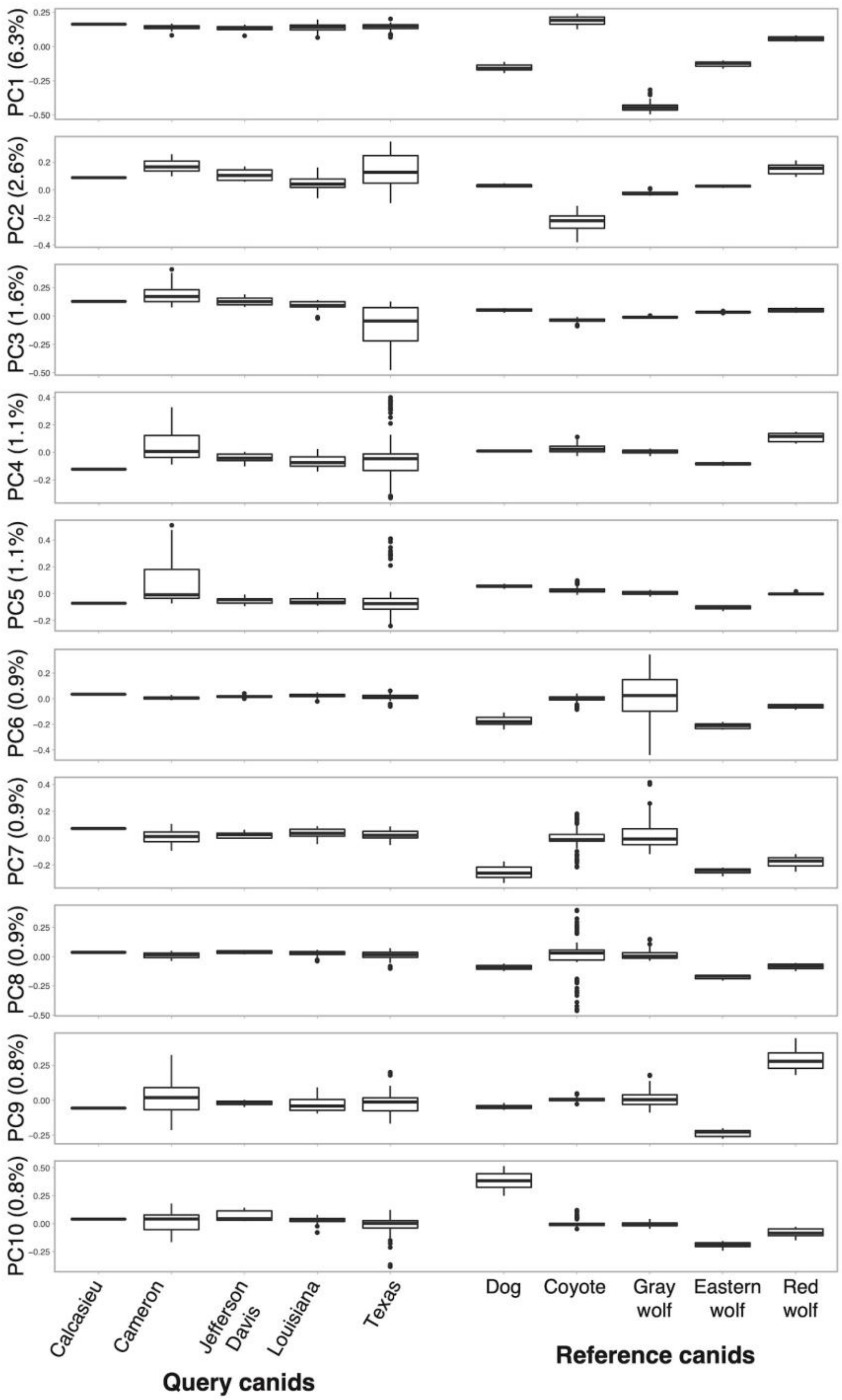

Figure S2. Histogram of the number of reads mapped to the canine Y chromosome for bioinformatic inference of sex. The solid line is the smoothed density curve.

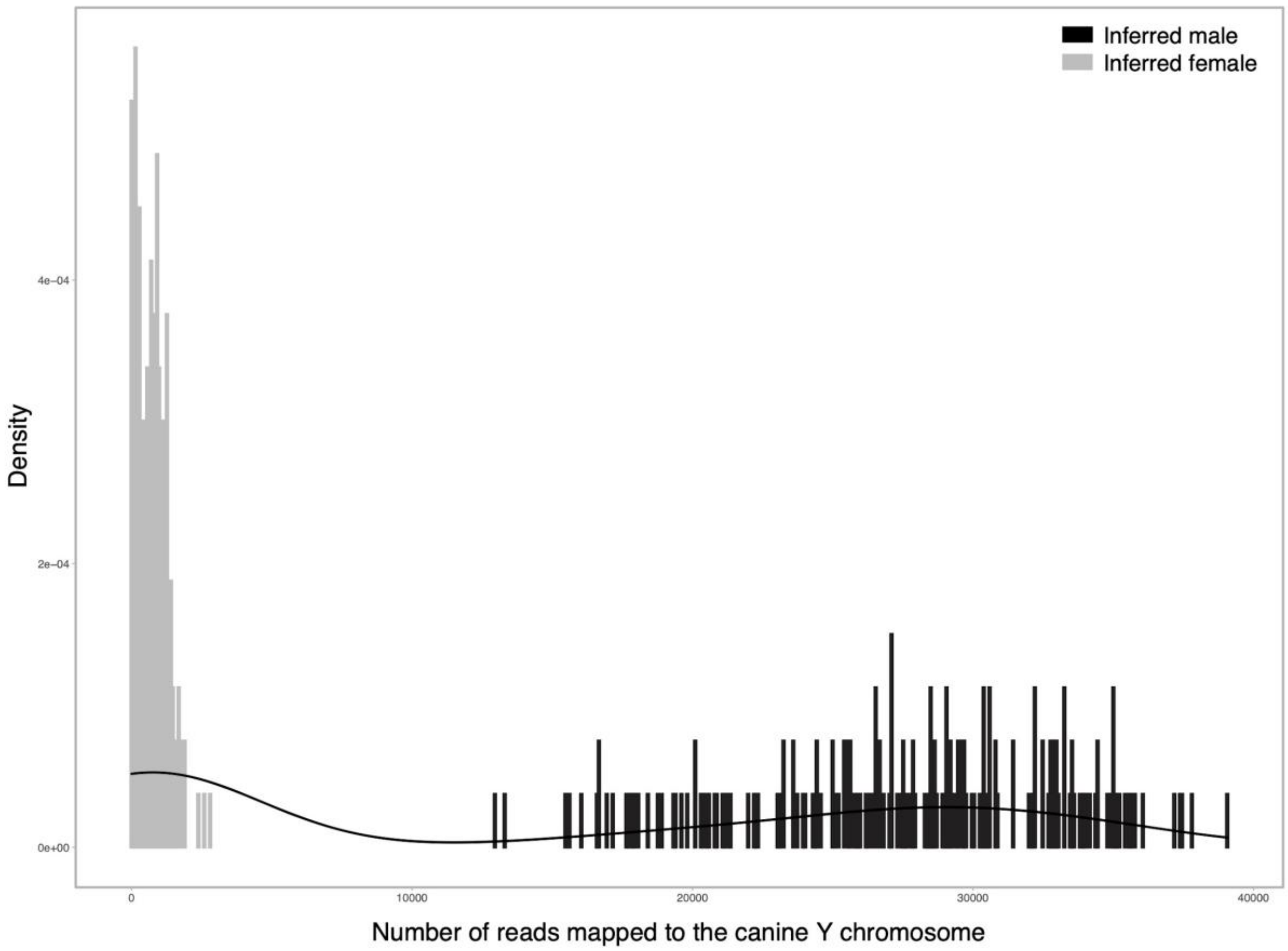

Figure S3. Comparison of phased autosomal chromosome copies of Red Wolf ancestry proportions with regression correlations for **A)** eastern Texas and **B)** southwestern Louisiana canids.

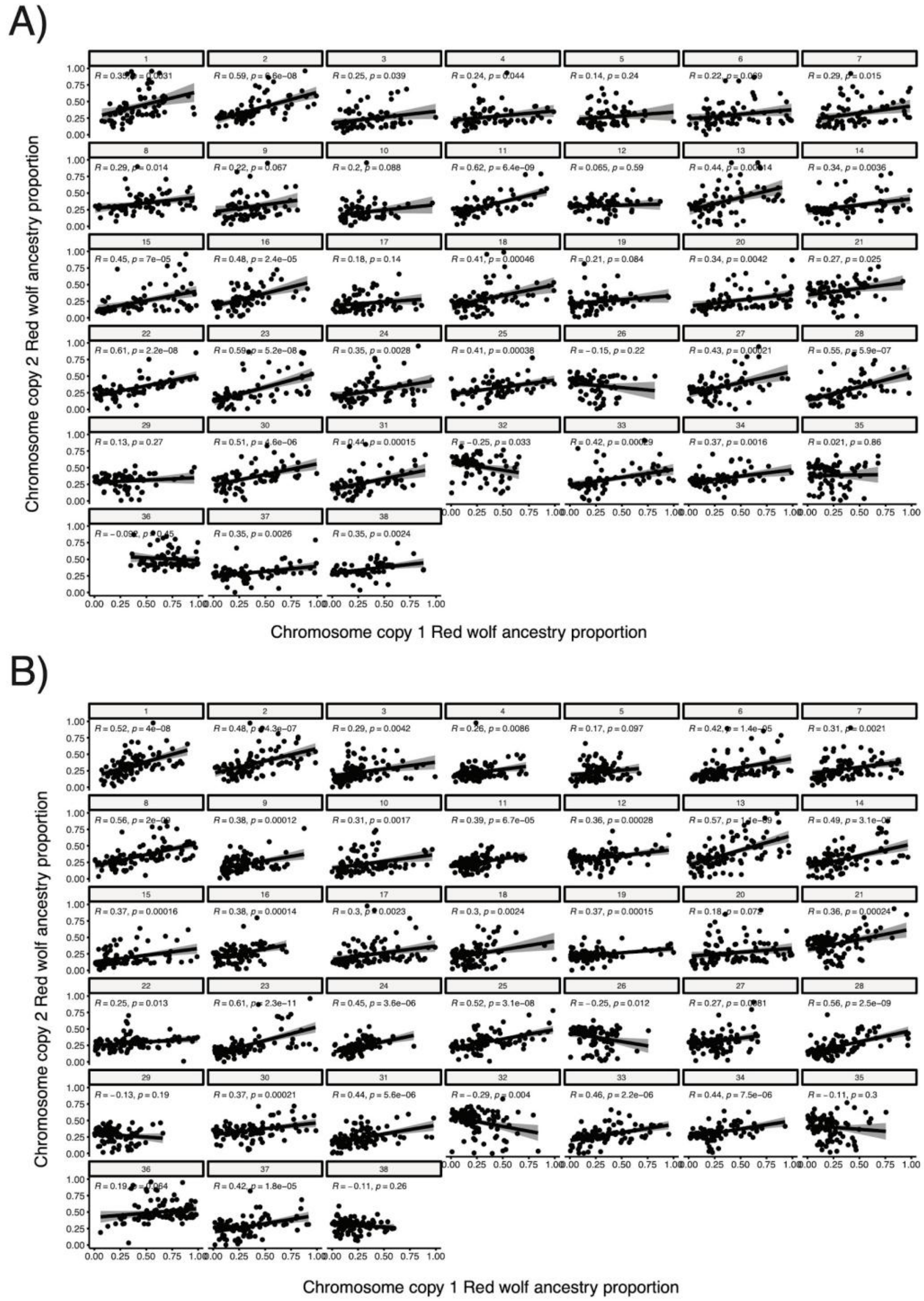

Figure S4. Distribution of absolute differences in global X chromosome-level Red Wolf ancestry for females. Pink shading indicates the density plot with the gray vertical bars composing the histogram.

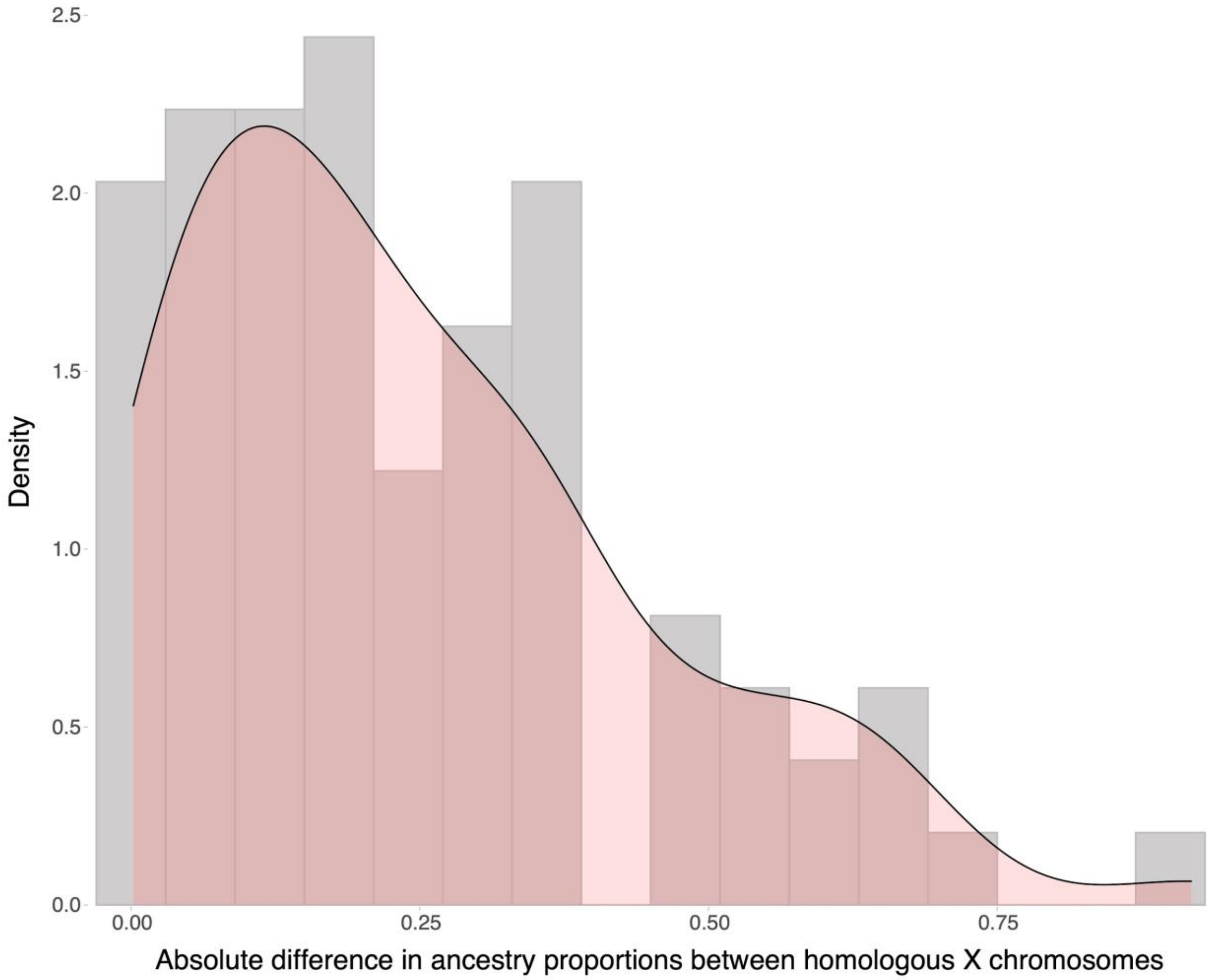

Supplement: Supplementary file 1 [file supplemental_figures_esag010.pdf]
